# Development of the Chick Microbiome: How Early Exposure Influences Future Microbial Diversity

**DOI:** 10.3389/fvets.2016.00002

**Published:** 2016-01-20

**Authors:** Anne L. Ballou, Rizwana A. Ali, Mary A. Mendoza, J. C. Ellis, Hosni M. Hassan, W. J. Croom, Matthew D. Koci

**Affiliations:** ^1^Prestage Department of Poultry Science, North Carolina State University, Raleigh, NC, USA; ^2^In Silico LLC, Fuquay-Varina, NC, USA

**Keywords:** chicken, microbiome development, *Salmonella*, probiotic, gut development

## Abstract

The concept of improving animal health through improved gut health has existed in food animal production for decades; however, only recently have we had the tools to identify microbes in the intestine associated with improved performance. Currently, little is known about how the avian microbiome develops or the factors that affect its composition. To begin to address this knowledge gap, the present study assessed the development of the cecal microbiome in chicks from hatch to 28 days of age with and without a live *Salmonella* vaccine and/or probiotic supplement; both are products intended to promote gut health. The microbiome of growing chicks develops rapidly from days 1–3, and the microbiome is primarily *Enterobacteriaceae*, but *Firmicutes* increase in abundance and taxonomic diversity starting around day 7. As the microbiome continues to develop, the influence of the treatments becomes stronger. Predicted metagenomic content suggests that, functionally, treatment may stimulate more differences at day 14, despite the strong taxonomic differences at day 28. These results demonstrate that these live microbial treatments do impact the development of the bacterial taxa found in the growing chicks; however, additional experiments are needed to understand the biochemical and functional consequences of these alterations.

## Introduction

Increasing evidence in multiple species demonstrate the impact gut microbes have on intestinal function, digestion, host metabolism, and immune function (1, 2). While the food animal industry has employed various methods to control and augment the bacteria in the gut for decades, this has been done with little understanding of the complexity of the microbial populations and their association with animal health. The advent of microbiome analysis will allow for better use of these products and the rational design of new therapies to promote animal health and performance. An estimated $585 million/year is spent globally on interventions to manage disease in food animals (3); many of these diseases are intestinal in nature (4), and the indirect costs of these intestinal diseases are far greater. The application of modern nucleotide sequencing and associated bioinformatics techniques to the avian gastrointestinal microbiome will lead to breakthroughs in our understanding of digestive processes, host metabolic regulation, immune function, and intestinal dysfunction and pathology. Collectively, increased understanding of the host–microbiome relationship, and the development of techniques to improve these interactions, could reduce the prevalence of food-borne pathogens. In order to effectively apply modern microbial ecology research techniques and elucidate the manner in which the avian intestinal microbiome interacts with the host genome, it is imperative to develop a comprehensive understanding of how the avian microbiome develops under different physiological states and management practices.

There is a dearth of information available on the development and definition of a normal avian gut microbiome. Recent investigations have begun to identify species commonly seen in adult chickens, but little is known about the intermediate and developing community (5–7). Furthermore, there is a paucity of information of the effects of treatments that target the gut environment on the development of the intestinal microbiome of chickens. This impairs our ability to understand how these gut-targeted treatments interact with each other and the host, and how they might affect gut activity and health. A better understanding of these interactions will allow for the rational use of bacterial groups to promote specific host responses.

The goal of this study was to understand the ontogeny of the chicken intestinal microbiome, and how commonly used live bacterial treatments influence this dynamic microbial community. Specifically, we included two live bacterial products currently used in the industry that are intended to improve animal health through manipulation of the host microbiota. We used a live attenuated *Salmonella enterica*, serovar Typhimurium vaccine (Salmune^®^, CEVA Biomune), and a probiotic feed supplement comprised of *Lactobacillus acidophilus, Lactobacillus casei, Enterococcus faecium*, and *Bifidobacterium bifidum*(PrimaLac^®^, Star Labs). We hypothesized that the species richness of the microbiome would increase rapidly, and the addition of live bacterial treatments would alter the development of microbial diversity and the composition of the microbiome. The results from this study demonstrated exposing developing chickens to individual or combined bacterial regimens leads to treatment-specific microbial populations. These populations continue to diverge with age, even in animals receiving only a one-time dose of the *Salmonella* Typhimurium (ST) vaccine at day of hatch. Predicted metagenomic content in these populations suggest changes in potential microbial metabolic activity and microbe-derived signaling molecules; however, these changes were less numerous than the taxonomic changes seen in the same populations.

## Materials and Methods

### Animals and Treatments

Two hundred one-day-old female commercial white leghorn laying type chickens (W-36, Hy-line International) were assigned to one of four treatments (50 chicks/treatment) in a 2 × 2 factorial design. The four groups were designated as follows: Diluent-Control (DC); Diluent-Probiotic (DP); Vaccine-Control (VC); and Vaccine-Probiotic (VP). Animals received either a one-time dose of a live, attenuated ST spray vaccine (Salmune^®^, Ceva Biomune, Lenexa, KS; Vaccine group) or a sham vaccination consisting of the vaccine diluent, water (Diluent group). The vaccine and diluent spray were administered as recommended by the manufacturer. These treatment groups were further divided into two dietary groups; one group (Control) was fed a standard corn–soybean starter diet (Table S1 in Supplementary Material) and the probiotic group was fed an identical starter diet supplemented with 0.1% (w/w) of the probiotic PrimaLac^®^ (*L. acidophilus, L. casei, E. faecium, B. bifidum*; Star Labs Inc., Clarksdale, MO, USA; Probiotic group). Probiotic pre-mix was added to the probiotic groups’ feed prior to the experiment and animals in all groups were fed *ad libitum* for 4 weeks.

Animals in all groups were housed in 934-1-WP isolators (L. H. Leathers Inc., Athens GA) climate-controlled HEPA-filtered isolation units. The animals were maintained and euthanized under an approved protocol from the North Carolina State University Institutional Animal Care and Use Committee (OLAW #A3331-01).

### Sample Collection

Six chickens from each treatment group were euthanized (CO_2_ followed by cervical dislocation) on days 0, 1, 3, 7, 14, and 28 and the contents of one cecal lobe collected and maintained on ice. At early timepoints, some chicks yielded minimal or no cecal digesta; these are noted in Table S2 in Supplementary Material. The cecal samples were weighed and diluted with 600 μl of 30% glycerol in PBS for storage at −80°C.

### DNA Isolation and 16S Sequencing

DNA was isolated from each cecal sample using the MO BIO Power Soil kit (MO BIO, Carlsbad, CA, USA) with the following modifications: a 10-min, 65°C incubation step was added and samples were then homogenized for 45 s at 5100 RPM using garnet bead-containing tubes and a Precellys 24 homogenizer (Precellys, Montigny-le-Bretonneux, France).

DNA recovered from the extraction process was quantified using a NanoDrop 2000 spectrophotometer (NanoDrop, Wilmington, DE, USA) and 10 ng from each sample were aliquoted into 96-well plates in a random order. Some animals contained only small amounts of cecal digesta, particularly at days 0–3, resulting in very small amounts of DNA for some samples. DNA from these samples was included in the sequencing process, despite the possibility of poor quality sequencing (Table S2 in Supplementary Material). MiSeq library preparation and 151 × 151 paired-end sequencing (Illumina, San Diego, CA, USA) were performed by the Argonne National Laboratory Institute for Genomics and Systems Biology Next Generation Sequencing Core using a protocol and primers recommended and previously described by the Earth Microbiome project and others. Primers used spanned the V4 region of the 16S rRNA gene (515F: GTG**Y**CAGCMGCCGCGGTAA, 806R: GGACTACHVGGGTWTCTAAT) (8). This primer set is commonly used to evaluate the microbiome community across a variety of fields, and is well validated in several models, including the chicken (8–11). Studies estimating microbial composition using V4 sequence information report diversity measurements comparable to those obtained with full-length 16S sequences (12).

### Sequence Data Analysis

The unpaired raw sequencing reads were paired and filtered using EA-Utils (13). Paired reads were processed using the QIIME suite of tools (v 1.8.0) (14); barcode matching and quality filtering were conducted prior to picking operational taxonomic units (OTUs). The 16S sequencing process did not yield equal sequence coverage for all samples, and some samples had very low sequence coverage. Samples with low sequence coverage or consistently poor quality were excluded from analysis. Additionally, some ceca from early time points contained little to no recoverable digesta. Consequently, a small number of samples from different time points were removed at this stage (Table S2 in Supplementary Material). OTUs were picked using an open-reference protocol. Briefly, sequences were grouped into OTUs based on 97% sequence identity using uclust and the Greengenes reference database (15, 16). OTUs that failed to match to the database were reclustered, resampled, and re-compared to the database; in this way, new reference sequences are compared to the database in order to minimize the number of excluded sequences. Finally, OTUs that failed to align to any sequences in the reference database are *de novo* clustered. Representative sequences from each OTU were picked and assigned taxonomy using the uclust consensus taxonomy assigner. During this process, sequences with high identity (>97%) were grouped into the same OTU, and are reported at the lowest level of taxonomic identification common to all sequences (17, 18). Sequence coverage was normalized across samples in each analysis. Taxonomic assignments, and alpha and beta diversity metrics were generated using QIIME and Primer-E (v6.1.16; Primer-E LTD, Ivybridge, UK). Principal coordinate analysis (PCoA) plots used in this study were generated in Primer-E using the Bray–Curtis distance metric (19).

Permutational multivariate analysis of variance (PERMANOVA) was conducted using the PERMANOVA+ add-on to Primer-E. Main and pair-wise tests were conducted using up to 1000 permutations of residuals under a reduced model. Similarity percentage analysis (SIMPER) of taxonomic groups between treatment groups and times was made in Primer-E using Bray–Curtis distances. Analysis of similarity (ANOSIM) tests were conducted using Primer-E. Tests were conducted using up to 1000 permutations and the Spearman rank correlation method. A global test statistic (R) was generated for each treatment; the rank similarities between and within treatments were calculated and compared. The global R statistic is a measure of the strength of a treatment group’s association with microbiome composition, with 1 being the strongest association and 0 being no association.

Metagenomic inferences from the 16S amplicon data were made using the QIIME suite of tools (14, 15, 17, 18), PICRUSt (20), and KEGG (21); statistics and visualization of functional data were depicted using STAMP (22). Closed-reference OTU-picking protocols were used to identify 16S sequences belonging to annotated genomes. Briefly, sequences were grouped into OTUs based on 97% sequence identity using uclust and the Greengenes reference database. Representative sequences from each OTU were picked and assigned taxonomy using the uclust consensus taxonomy assigner. PICRUSt and KEGG were used to generate a list of functional genes predicted to be present in the sample and to organize these genes into gene pathways. Using STAMP, heatmaps were generated displaying differences in gene-group abundance at each time point. In order to minimize the number of treatment-based differences that may not be biologically relevant, analysis was limited to those differences with an effect size greater than 0.7 as calculated by STAMP (eta-squared method) (22). Storey’s FDR correction was applied to all comparisons between treatments (23). Nearest neighbor hierarchical clustering was used to group each sample according to abundance of gene groups in question.

## Results

### Microbiome Composition and Complexity Change Rapidly with Age

**Figure 1:**
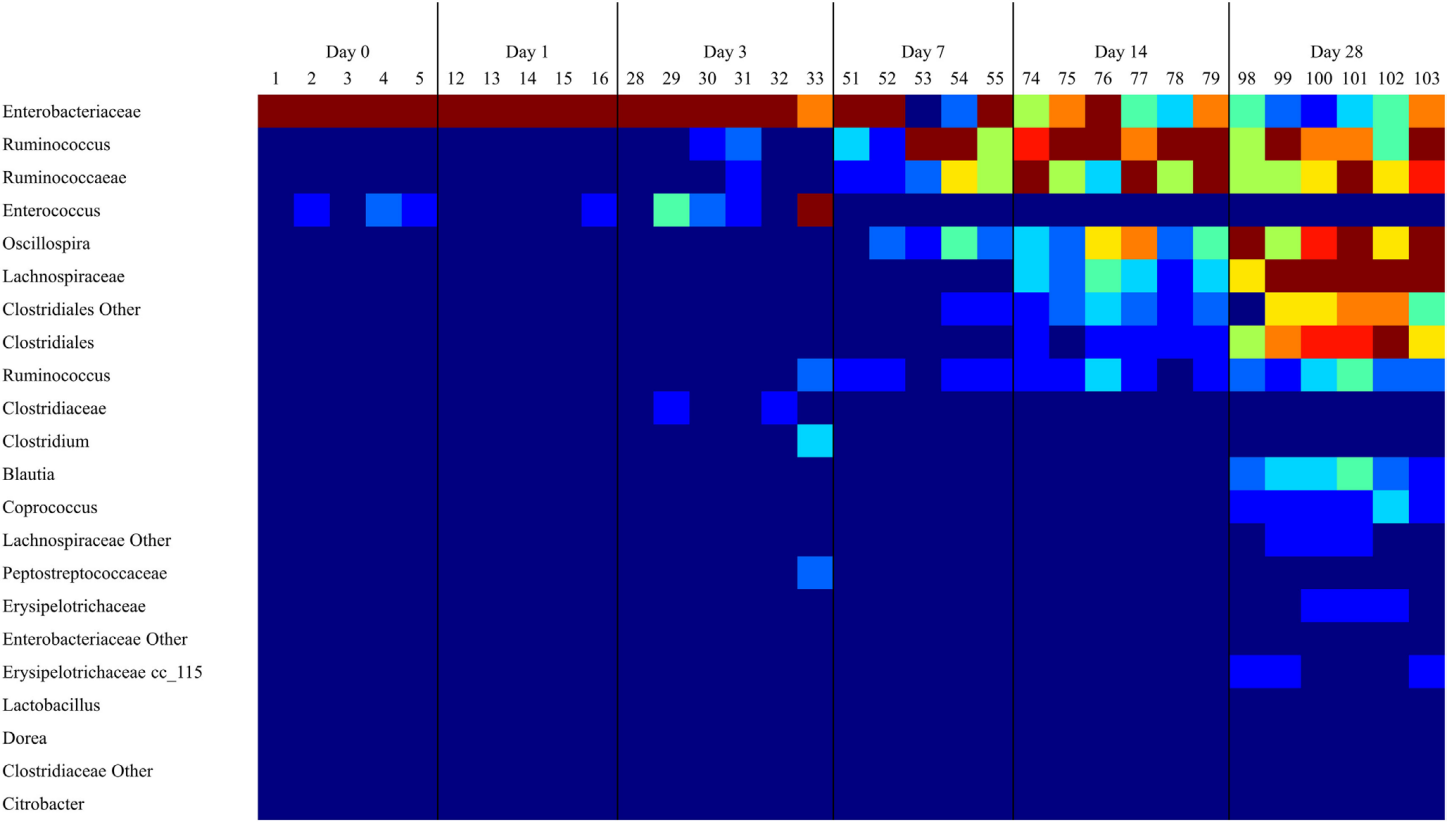
**As the cecal microbiome develops, the dominant taxa shift from Gram-negative to Gram-positive bacteria**. A heatmap of taxonomic groups present in untreated (DC) samples over time was generated with Qiime. The composition of the microbiome in DC animals was evaluated to identify trends in the development of the normal microbiome over time. There is a consistent decrease in the proportion of *Enterobacteriaceae* and *Enterococcus* over time, and an increase in levels of *Clostridiales* groups like *Ruminococcus* and *Oscillospira*. Sequence coverage was normalized to 16,260 reads/sample.

16S rRNA sequence analysis of the microbiome from the ceca of untreated animals (DC) demonstrated a microbiome with low diversity in days 0 and 1, dominated by *Enterobacteriaceae* and to a lesser extent *Enterococcus* (Figure 1). The number of OTUs detected in the microbiome increased significantly (*P* < 0.05) by day 3 (data not shown). This increase in bacterial richness starts with *Ruminococcaceae* groups during the first week of life and continues with other *Firmicutes*. By day 14, and extending through day 28, *Ruminococcus* and other *Firmicutes* outnumber *Enterobacteriaceae* (Figure 1).

### Age More Influential in Microbiome Development than Treatment

**Figure 2:**
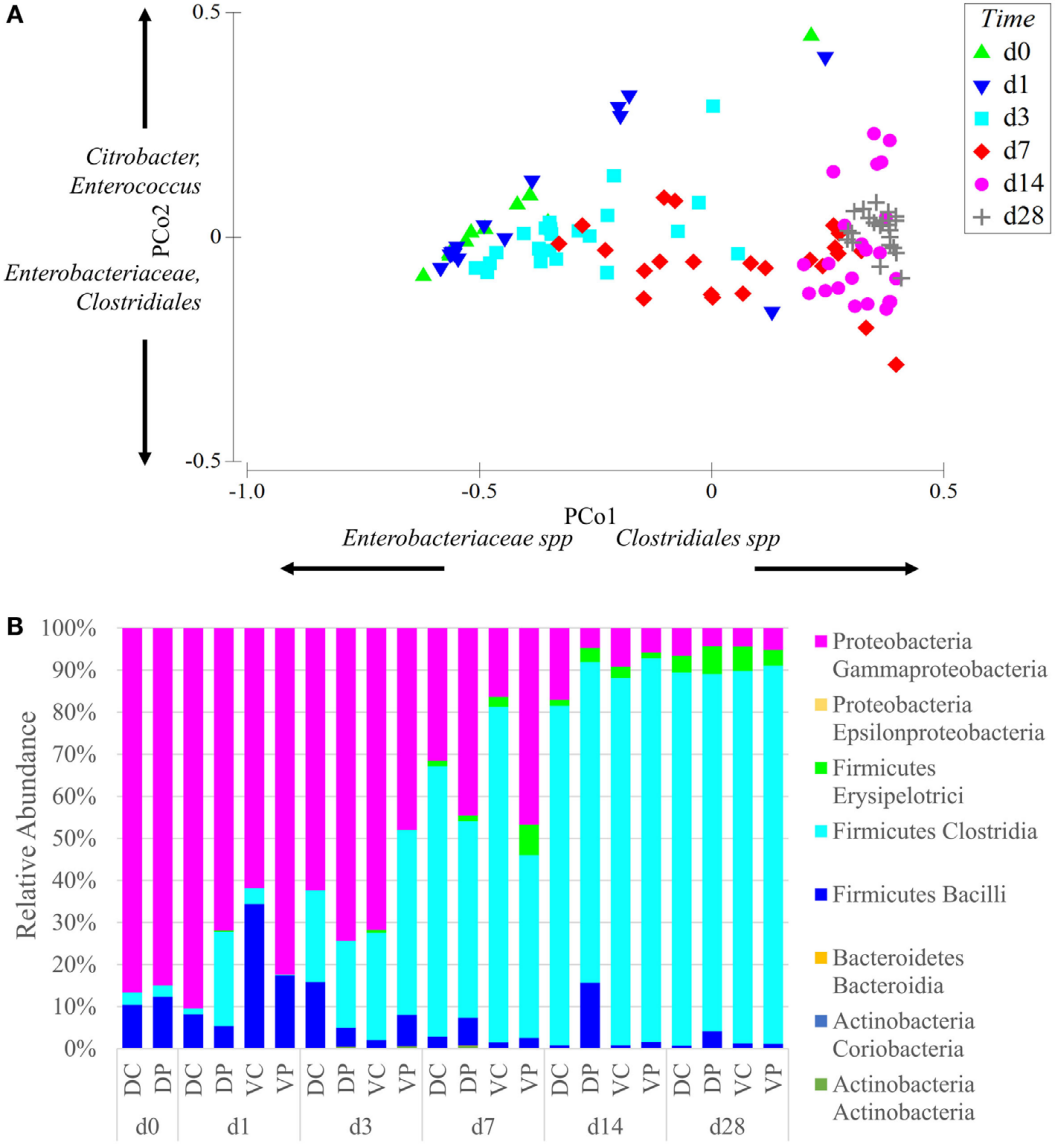
**Age is the dominant factor in the composition of the microbiome**. **(A)** Principal coordinate analysis of samples was conducted using Primer-E and samples were labeled based on age. Samples are clustered on two axes based on a multi-dimensional analysis of their sequence diversity and abundance. As the animals age, their microbiome increases in complexity but decreases in variability between samples, even between treatment groups. The effect of age was stronger than the effect of vaccine or probiotic. Differences between all time points were significant at permutational *P*-value <0.05, with the exception of day 0 vs. days 1 and 3. Coordinate loading for each principal coordinate shows the primary taxonomic groups contributing to each axis. Each data point represents a sample in the appropriate time point, and samples from all treatment groups are included in the analysis. **(B)** The phylum and class of sequences with an average relative abundance of 1% or greater are displayed by time point and treatment. All treatment groups started with high levels of *Gammaproteobacteria* that shifted with age into a *Firmicutes-*dominated community with large numbers of *Clostridia*. Taxonomy assignments were generated with QIIME, and PCoA plots were generated with Primer-E. Sequence coverage was normalized to 16,260 reads/sample.

Principal coordinate analysis of samples across all time points and treatment groups reveals that the effect of animal age on community composition was larger than that of bacterial treatment (Figure 2A). The ANOSIM-generated global test statistics for time (*R* = 0.67) and the treatments (Vaccine *R* = 0.361, Probiotic *R* = 0.317) demonstrate the relative impact of each on the community. At time points 0–7, the samples show large within time point variability. At day 28, within time point variability is decreased and samples are tightly clustered in the PCoA plot (Figure 2A). Community analysis of cecal samples across time points and treatment groups show that Gram-negative bacteria (*Proteobacteria*) dominate at early time points, while Gram-positive *Firmicutes*, especially *Clostridia* taxa, become more prominent with age (Figure 2B).

### Treatments Alter Microbial Composition and Rate of Development

**Figure 3:**
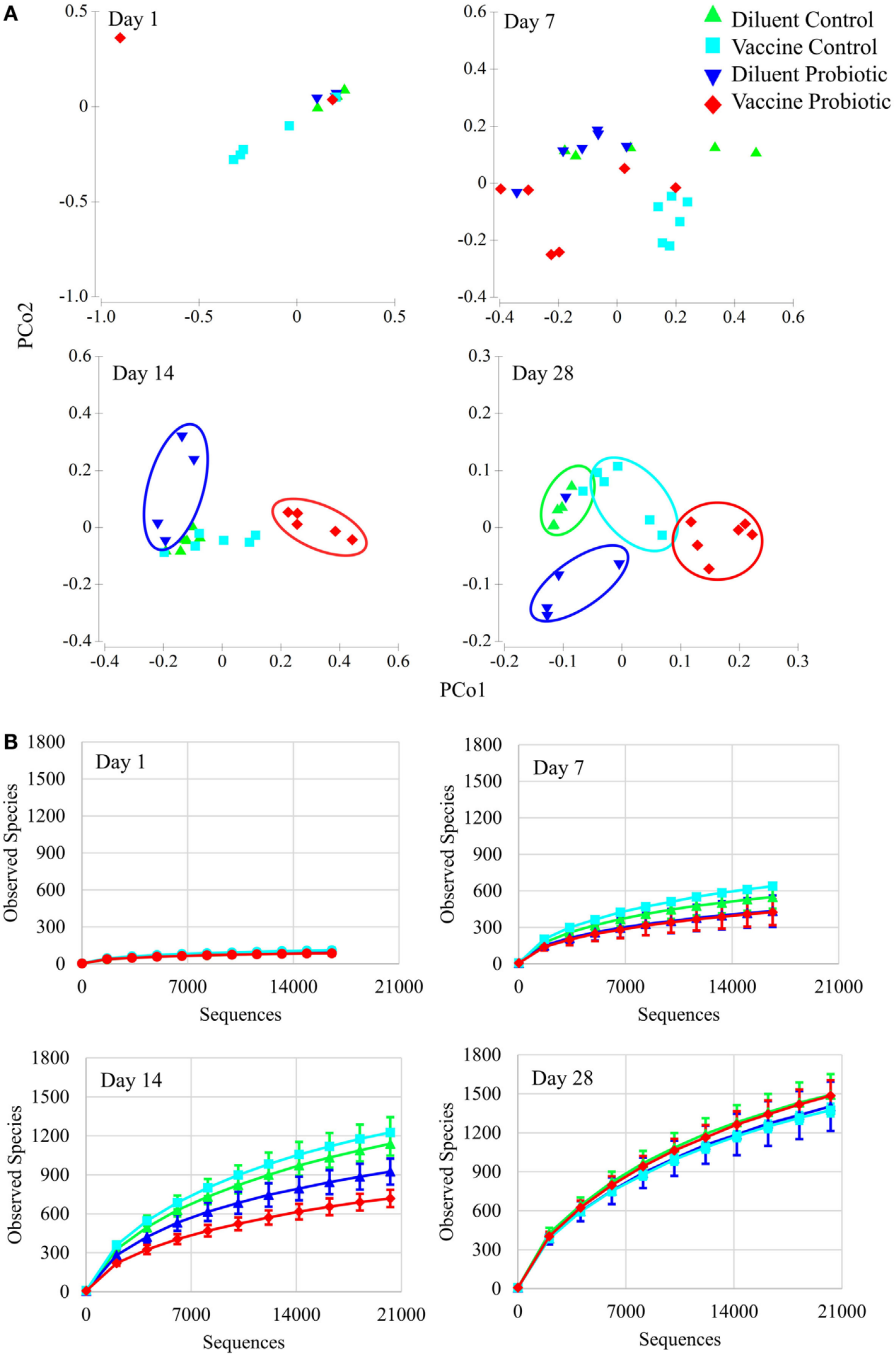
**Principal coordinate analysis and rarefaction analyses demonstrate the impact of the treatments over time**. **(A)** Principal coordinate analysis generated with Primer-E demonstrates the effect of treatments at 1, 7, 14, and 28 days of age. There are no significant treatment differences at day 1. By day 7, treatment groups are statistically different based on PERMANOVA tests. Treatment groups cluster visually at days 14 and 28. All treatment groups at days 7, 14, and 28 were different at permutational *P* < 0.05. **(B)** Rarefaction of observed species (unique OTUs) at individual time points was conducted using QIIME, and demonstrate the rapid development of taxonomic diversity. Treatment groups show similar diversity at Day 1. At day 7, DP and VP tend to have lower diversity than VC (*P* = 0.078 and 0.054, respectively). At day 14, DP and VP diversity is significantly lower than DC and VC diversity (*P* < 0.05). By day 28, community diversity is similar between treatments. Sequence coverage was normalized for each time point individually: day 1 (16,577 reads/sample), day 7 (16,668 reads/sample), day 14 (20,263 reads/sample), and day 28 (20,263 reads/sample).

Analyses of microbial populations were conducted within each time point (days 1, 7, 14, and 28) to assess the impact of treatment on composition and richness of the microbiome independent of age. No differences in microbial composition were detected at day 1, but significant differences in cecal microbiome composition were observed among all four treatment groups by day 7 (Figure 3A). A PERMANOVA showed that all four treatment groups are distinct in composition at days 7, 14, and 28 (*P* < 0.05). A comparison of taxonomic richness (alpha diversity) among treatment groups at days 1, 7, 14, and 28 was made using rarefaction plots. The treatment groups show similar levels of unique taxa at day 1; however, at days 7 and 14, probiotic groups tend to have fewer unique taxa (*P* < 0.1 at day 7, *P* < 0.05 at day 14). Interestingly, there was no significant difference in alpha diversity at day 28 (Figure 3B).

### Treatment with Live Bacteria Affects Abundance of Taxa not Associated With Treatment

Similarity percentage analysis conducted between treatment groups at days 14 and 28 indicates that the differences between treatment groups can largely be attributed to changes in the most abundant order, *Clostridiales*, including *Lachnospiraceae* and *Ruminococcaceae* genera (Tables 1 and 2). MANOVA was used to identify differentially abundant taxa between treatment groups, with an FDR correction made to account for multiple comparisons. At day 14, DC animals harbored a significantly higher proportion of *Enterobacteriaceae* (Table 1) with 16% *Enterobacteriaceae* as compared to 3–9% in the other treatments. *Lactobacillus* was significantly increased in the DP group relative to DC. At day 14, 83% of significantly different taxa were *Firmicutes*, and 63% were *Clostridia*.

**Table 1:** **Similarity percentage analysis (SIMPER) of treatment groups at day 14^a^**.

Phylum	Class	Order	Family	Genus	Average abundance (%)		% Contribution to dissimilarity^b^
					DC	DP	
Firmicutes	Bacilli	Lactobacillales	Lactobacillaceae	Lactobacillus	1	16*	19.4
Proteobacteria	Gammaproteobacteria	Enterobacteriales	Enterobacteriaceae^c^		16	3*	15.65
Firmicutes	Clostridia	Clostridiales	Lachnospiraceae	Ruminococcus	25	26	15.27
Firmicutes	Clostridia	Clostridiales	Ruminococcaceae		19	14*	9.01
Firmicutes	Clostridia	Clostridiales	Ruminococcaceae	Oscillospira	11	15	8.58
					**DC**	**VC**	

Firmicutes	Clostridia	Clostridiales	Lachnospiraceae		8	16*	15.4
Firmicutes	Clostridia	Clostridiales			4	12*	14.57
Firmicutes	Clostridia	Clostridiales	Ruminococcaceae		19	13*	13.61
Firmicutes	Clostridia	Clostridiales	Lachnospiraceae	Ruminococcus	25	24	13.28
Proteobacteria	Gammaproteobacteria	Enterobacteriales	Enterobacteriaceae		16	9*	13.12
					**DC**	**VP**	

Firmicutes	Clostridia	Clostridiales	Lachnospiraceae		8	39*	25.58
Firmicutes	Clostridia	Clostridiales	Lachnospiraceae	Ruminococcus	25	2*	18.69
Firmicutes	Clostridia	Clostridiales	Ruminococcaceae		19	3*	13.46
Proteobacteria	Gammaproteobacteria	Enterobacteriales	Enterobacteriaceae		16	5*	9.07
Firmicutes	Clostridia	Clostridiales			4	14*	8.95

*^a^Top 5 taxa shown for each comparison*.

*^b^Percent contribution to total dissimilarity between treatment groups under comparison*.

*^c^If a sequence matches more than one possible taxon, classification stops at the next highest level*.

*Indicates significance at *P* ≤ 0.05.

**Table 2:** **Similarity percentage analysis (SIMPER) of treatment groups at day 28^a^**.

Phylum	Class	Order	Family	Genus	Average abundance (%)		% Contribution to dissimilarity^b^
					DC	DP	
Firmicutes	Clostridia	Clostridiales	Lachnospiraceae	Ruminococcus^c^	12	19	16.65
Firmicutes	Clostridia	Clostridiales	Ruminococcaceae		11	18*	12.94
Firmicutes	Clostridia	Clostridiales	Other	Other^d^	11	5*	9.57
Firmicutes	Clostridia	Clostridiales	Ruminococcaceae	Oscillospira	12	8*	9.22
Firmicutes	Clostridia	Erysipelotrichales	Erysipelotrichaceae		2	5	7.73
					**DC**	**VC**	

Firmicutes	Clostridia	Clostridiales			12	21*	15.41
Firmicutes	Clostridia	Clostridiales	Lachnospiraceae		15	10*	13.49
Firmicutes	Clostridia	Clostridiales	Other	Other	11	4*	12.26
Firmicutes	Clostridia	Clostridiales	Ruminococcaceae	Ruminococcus	5	9	7.53
Firmicutes	Clostridia	Clostridiales	Lachnospiraceae	Ruminococcus	12	12	7.45
					**DC**	**VP**	

Firmicutes	Clostridia	Clostridiales			12	36*	32.27
Firmicutes	Clostridia	Clostridiales	Lachnospiraceae		15	5*	14.03
Firmicutes	Clostridia	Clostridiales	Other	Other	11	1*	13.05
Firmicutes	Clostridia	Clostridiales	Ruminococcaceae		11	16*	6.85
Firmicutes	Clostridia	Clostridiales	Lachnospiraceae	Ruminococcus	12	11	5.11

*^a^Top 5 taxa shown for each comparison*.

*^b^Percent contribution to total dissimilarity between treatment groups under comparison*.

*^c^If a sequence matches more than one possible taxon, classification stops at the next highest level*.

*^d^“Other” indicates the sequence in question has not been assigned to a taxonomic group at that level*.

*Indicates significance at *P* ≤ 0.05.

Analysis of the taxonomic groups represented in each treatment at day 28 indicate that most significant changes occur in the *Firmicutes* phylum, including differences in the abundance of *Ruminococcaceae, Lachnospiraceae*, and *Peptostreptococcaceae* (Table 2). *Lactobacillus* is also increased in the DP group relative to DC. Eighty-one percent of all significantly different taxa at day 28 were in the Order *Clostridiales*.

### Treatment-Induced Changes in Microbiome Diversity Lead to Predicted Changes in Abundance of Functional Gene Families

**Figure 4:**
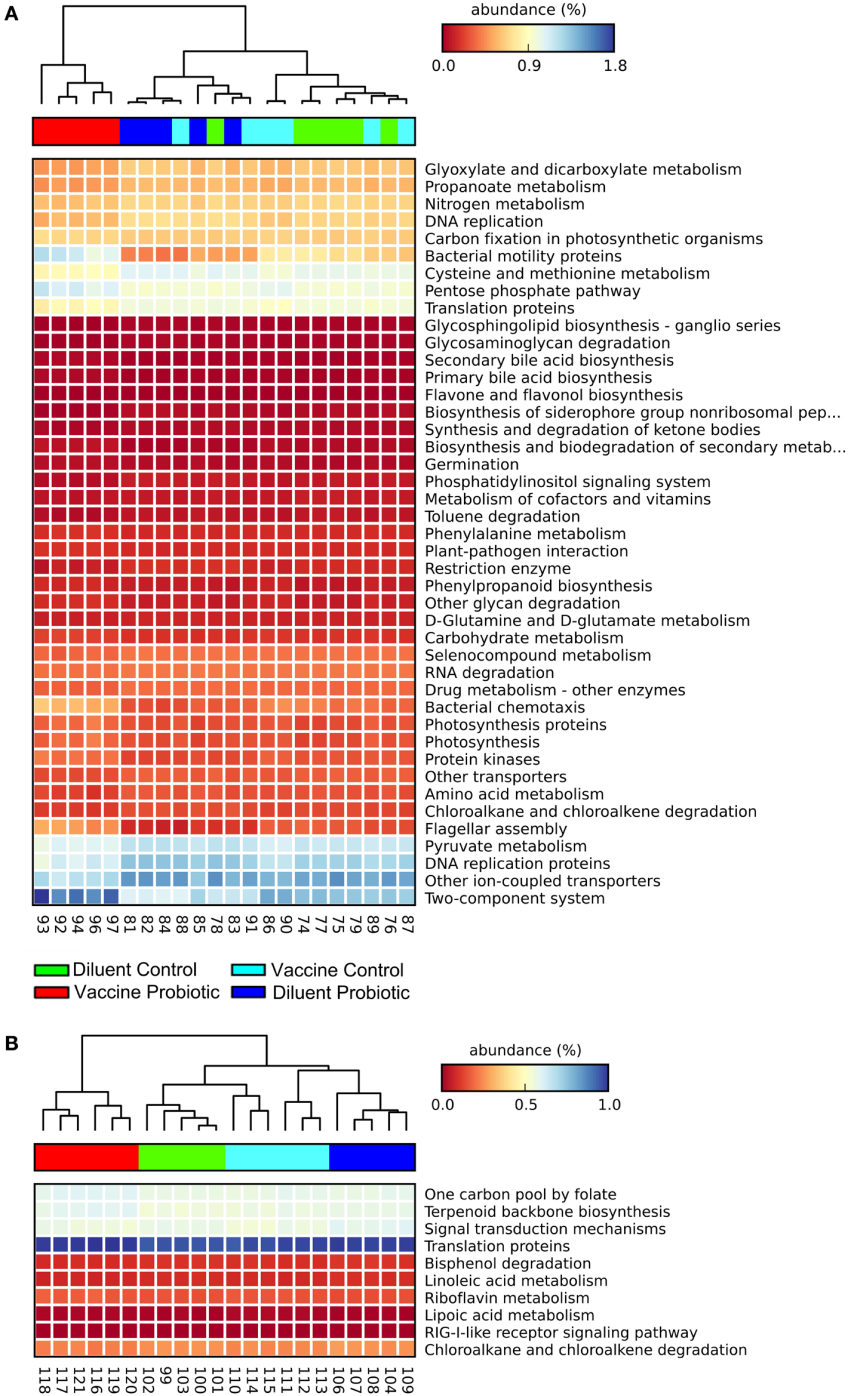
**At both days 14 and 28, the VP group shows the greatest divergence in predicted metagenomic content**. PICRUSt was used to generate a list of genes inferred to be present in the samples, their relative abundance, and the gene pathways with which they are associated. A heatmap was generated with STAMP. Samples were clustered using a nearest neighbor metric, and pathways were colored based on their percent abundance relative to all measured genes. All listed gene groups are significantly different between treatment groups with an effect size >0.70 and FDR-corrected *P* < 0.01. The combination of vaccine and probiotic treatments stimulates changes in several gene groups and pathways at day 14 **(A)**, namely increases in chemotaxis, two-component system, flagellar assembly, and bacterial motility. Decreases in the VP group include metabolic processes, such as amino acid metabolism, DNA replication, and protein translation. **(B)** There are fewer significantly different pathways at day 28 and changes are largely related to cell metabolism.

Estimates were made of the functional changes that may occur in the cecal microbiome following treatment using closed-reference OTU-picking and PICRUSt. Gene groups targeted for statistical analysis had an FDR-corrected *P* < 0.01, and an effect size of 0.7 or higher. At day 14, samples cluster primarily by probiotic treatment, and the VP group is most distinct from other treatments. The combination of ST and probiotic treatments increases the expected proportion of genes related to environment-sensing; two-component systems, bacterial motility, chemotaxis, and flagellar component assembly genes were predicted to increase. DC, DP, and VC groups have relatively higher abundance of genes related to amino acid metabolism, DNA repair and replication, and translation (Figure 4A). The differences between DC, DP, and VC groups were minor, but the DP group displayed the lowest expected abundance of two-component system and bacterial motility genes.

Fewer gene groups met the inclusion criteria at day 28, and the total relative abundance of included gene groups was lower than that at day 14. At day 28, DP and VP treatment groups display higher predicted levels of genes related to one carbon metabolism, terpenoid synthesis, and translation proteins (Figure 4B). DC and DP groups had higher proportions of fatty acid metabolism, drug metabolism, and signal transduction gene pathways.

**Figure 5:**
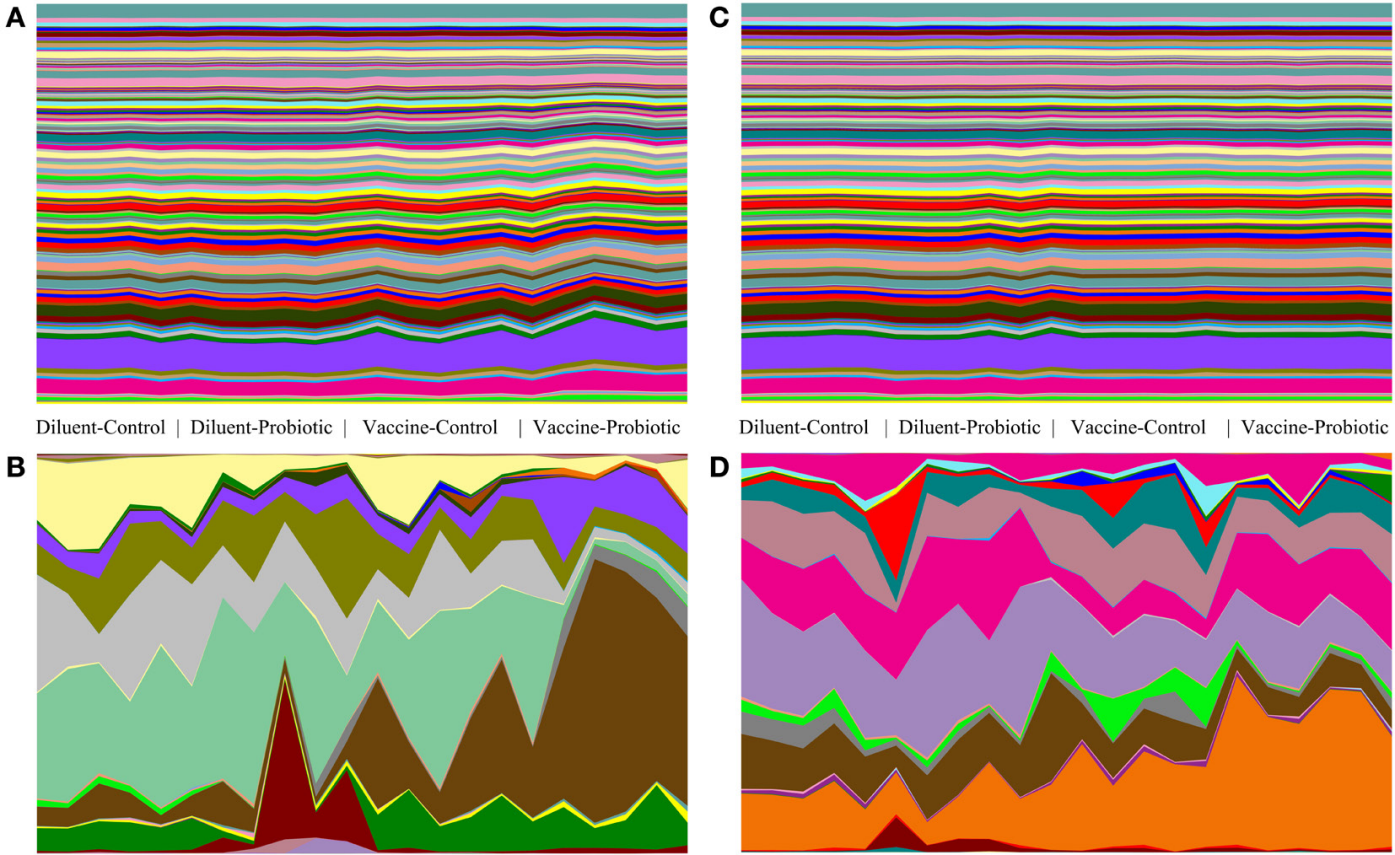
**Comparison of taxonomic and gene-group abundance trends at days 14 and 28**. Relative abundance of taxonomic groups and predicted abundance of functional gene groups demonstrate the stability of gene-group abundances relative to changes in taxonomic groups. Relative abundance tables for taxa and gene group were assembled and used to generate area charts of all samples at days 14 and 28. The relative abundance of every identified taxonomic or functional gene group is shown for each sample; **(A)** predicted gene groups at day 14, **(B)** taxonomic groups at day 14, **(C)** predicted gene groups at day 28, **(D)** taxonomic groups at day 28. At day 14, there are clear taxonomic differences between treatments **(A)**, and smaller changes in the abundance of a few gene groups **(B)**. At day 28, taxonomic changes **(D)** are accompanied by few visible changes in gene-group abundance **(C)**.

Relative abundance tables of taxa and predicted gene groups were used to generate area charts of between-treatment changes in taxa and gene groups. Vaccine and Probiotic groups differ from the DC group taxonomically at both days 14 and 28. Gene-group abundance shows less treatment-specific variability (Figure 5).

## Discussion

Little is known about the development of the microbiome in young birds, and how it is affected by different stimuli (7). The goal of this study was to characterize the healthy developing microbiome in chickens and understand how commonly used bacterial treatments intended to improve or maintain health would affect this process. This is important in the food animal industry as there are numerous feed additives intended to improve animal health, either directly or indirectly through improving gut health. However, the mechanisms by which these amendments work are poorly understood. Most claim to enhance health and performance via manipulation of the host intestinal microbiome, but the mechanism of action has been studied in very few of these products (24, 25).

In the present study, we administered two commonly used live bacterial treatments applied in poultry production to enhance intestinal health and function. According to the manufacturer, the live ST vaccine used here is intended to prevent colonization of the gut and internal organs by multiple types of *Salmonella*, including Heidelberg, Typhimurium, Hadar, Kentucky, and Enteritidis (26). Similarly, the probiotic used here is intended to maintain healthy microbiota balance in the gut (27). We investigated to what extent these health-promoting treatments affect the microbiome of young chicks.

We found that the post-hatch intestinal microbiome has low diversity dominated by Gram-negative bacteria, particularly *Enterobacteriaceae*, which includes *Salmonella, Klebsiella, Proteus*, and *Escherichia coli*. During the first week of life, there is a shift to a much more diverse community comprised of a wide variety of Gram-positive bacteria, mainly within the *Clostridiales* group, resulting in a correspondingly smaller proportion of Gram-negative bacteria (Figure 1). The proportion of Gram-negative bacteria in the cecum at day 28 is <6%, and it is almost entirely *Enterobacteriaceae*.

Data from the present study suggest a microbiome more affected by age than treatment (Figure 2A). Irrespective of treatment, all groups show a sharp decline in *Enterobacteriaceae* with age, including the vaccinated groups, where levels of *Enterobacteriaceae* would be expected to increase following *Salmonella* (member of the *Enterobacteriaceae* family) vaccination. Nor does addition of a *Firmicutes-*based probiotic product stimulate more rapid conversion to a *Firmicutes*-dominated microbiome (Figure 2B) in probiotic-fed animals. Day-old birds begin with a gut colonized by few bacterial species at a concentration several orders of magnitude lower than mature animals (28, 29), so it is likely that the primary driver of age-dependent increase in complexity is bacteria colonizing a previously empty niche. However, diet can also play a major role in the composition of the microbiome and exerts an influence on the developing and mature gut (30, 31). Studies by Sergeant et al. characterizing the microbiome of wheat-fed chickens reported *Megamonas* and *Negativicutes* as more abundant in their adult birds, while the *Firmicutes* most commonly seen in this trial, *Lachnospiraceae* and *Ruminococcaceae*, were less abundant (32). The effect of gut development on the intestinal microbiome is more difficult to quantify; though studies of germ free and gnotobiotic mice clearly demonstrate that the microbiome is essential to the development of a fully functioning gut (33, 34), whether the developmental stage of the gut is a variable influencing the development of the microbiome is less clear.

Despite the strong relationship between age and composition of the microbiome, the bacterial treatments included in this study did affect the microbiome. PCoA of the four treatment groups at days 1, 7, 14, and 28 illustrate the impact of both vaccination and probiotic supplementation on the microbiome (Figure 3A). Despite the fact that the vaccine is only administered on day 0, global R statistics demonstrate that the impact of ST on the composition of the microbiome is on par with that of the continuously fed probiotic at days 14 (vaccine = 0.802, probiotic = 0.882) and 28 (vaccine = 0.697, probiotic = 0.705). The magnitude of the effect of the one-time ST inoculation is nearly as great as that of the continuously fed probiotic despite low levels of the ST-containing taxonomic group *Enterobacteriaceae* after day 7, suggesting early colonizers influence the relative abundance of the microbiome despite being transient themselves. While little is known about the long-term effects of early microbiome perturbation, some studies support this idea (35, 36).

To understand the impact of treatment at the taxonomic level, SIMPER was conducted to identify species contributing to differences between treated and untreated animals. Most of the differences between DC and treated animals at days 14 and 28 involve *Lachnospiraceae, Ruminococcaceae*, and other *Clostridiales* (Tables 1 and 2). Though abundance of *Lactobacillus* in the DP and VP groups is higher at day 14 (*P* < 0.05), the magnitude of the increase in VP over DC is not great enough for *Lactobacillus* to be a major source of dissimilarity. There is little microbiological evidence that the bacterial products applied in this study interfere with each other; levels of *Lactobacillus* are not significantly lower in the VP group than the DP group, and VP animals remain ST-positive at day 28 (data not shown). There are signs of treatment interaction; however, ST vaccination decreases abundance of the group *Clostridiales Other*, and the combination of probiotic and vaccine results in the strongest difference from the DP group; 11% reduced to 1% of the identified bacteria, perhaps indicating a synergy between the two treatments, which makes the cecum a more hostile environment for this group of bacteria.

Changes in taxonomic diversity are the most used indicator to infer changes in microbiological activity, but it is becoming apparent that many of the functions of a normal microbiome can be carried out by a number of microbial groups (37, 38). Therefore, understanding how treatments affect taxonomic abundance may not provide us with a complete understanding of how they impact healthy and diseased guts, or develop therapies that target the predominant cause of gut dysbiosis; a change in function. In its entirety, the chicken gut is estimated to be colonized by as many as 10^13^ microbes, and they have a combined genetic potential far in excess of the ~20,000 genes identified in the chicken genome (39, 40). PICRUSt uses the 16S rRNA genes obtained during sequencing to infer the presence of functional genes known or predicted to be associated with those 16S sequences. At day 14, there were predicted increases in the VP group in genes related to motility, flagellar assembly, chemotaxis, and two-component system. By contrast, VP microbiomes displayed lower abundance of many protein and energy metabolism genes, as well as genes related to DNA replication and protein translation (Figure 4A). Supporting the taxonomic data suggesting that the microbiome is still equilibrating at day 14 (Figures 2 and 3), the functional changes at day 28 are both fewer and less dramatic (Figure 5). The VP group exhibits the most variation of the four treatments, and suggests changes in a few cellular metabolism pathways. Interestingly, the effect of probiotic supplementation and its interaction with the vaccine appears to stimulate more functional changes than the vaccine group alone. At days 14 and 28, the DP and VP groups were more likely to have either the highest or lowest levels of any given gene group.

A possible contributor to the lack of more dramatic functional diversity between treatment groups at day 28 could be limitations inherent to this technique and its application to avian microbial communities. One of these is its reliance on sequenced and annotated genomes. Though comparisons between PICRUSt results and metagenomics data from the same samples have shown that the predictive value of PICRUSt analysis is very good (20, 41), 16S genes without a confident phylogenetic assignment cannot be used as marker genes. Because of this, about 15% of the 16S sequences were filtered out at day 14 and over 20% at day 28. This number of unknown or uncharacterized sequences may be higher in the avian microbiome than in the human or murine microbiome, as the databases used in this process were all developed based on mammalian microbiota; chicken-specific microbes that may be important in this system could be excluded from analysis because they are not part of the 16s and/or KEGG databases. However, this analytical technique has been successfully used on avian microbiomes in the past (6). While the difference in excluded taxa between time points is not large, it is possible that the bacteria excluded from analysis are active in the community; evaluation of those taxa excluded from PICRUSt analysis indicates that some are differentially abundant between treatment groups (Tables S3 and S4 in Supplementary Material). These bacteria could play a significant role in the activity of the microbiome. Bacteria falling under the *Clostridiales Other* group were consistently higher in the DC group relative to other treatments, and could represent an unmeasured source of functional differences between treatment groups. However, their metagenomic contribution to the community cannot be known without further characterization of their genome.

The relative lack of functional gene differences at day 28 could also be an indication that despite continuing taxonomic differences, the microbiome in each treatment is converging toward a similar metabolic pattern. Conservation of function across a variety of microbial profiles has been described in other studies, and extreme dysregulation of the microbiome may be required before severe or protracted functional changes occur (37). Figure 5 illustrates this concept; while the bacterial treatments applied in this study affect both the taxonomic and inferred metagenomic composition of the microbiome, even statistically significant changes in function gene content are minor when compared to the taxonomic changes seen in the same animals.

In the present study, the chickens were all free of visible disease or stress, and it is possible their gut microbiota were functioning in their optimal range with or without treatment. It is also important to note that these birds were not given a pathogen challenge or other stressor of any kind. The addition of vaccinations or probiotics to a chicken with a dysbiotic gut microbiome might yield more significant functional changes. Recent studies demonstrated that exposure of mice to antibiotics at an early age can have a deleterious effect on the diversity of the microbiome for several months following treatment (42). This study showed no such effect from probiotic or vaccination. The value of select dietary treatments and management practices in poultry production may be their ability to increase the speed at which a disturbed or stunted microbiome is able to return to a normal functional state.

In conclusion, one-time oral inoculation with a live ST strain and daily ingestion of a probiotic feed supplement both alter the microbiome of growing chicks. These differences persisted throughout the study, and are centered on changes in the abundance of core microbes present in all treatment groups. The results of this trial suggest that common bacterial treatments, such as probiotics and bacterial vaccines, affect the taxonomic composition of the microbiome, but only have transient or small effects on the function and activity of the microbiome under non-stressed growth conditions. By contrast, as has been seen in other studies (7), age played a major role in the composition and richness of the bacterial community. Major shifts from day of hatch to day 14 centered on the early dominance of *Enterobacteriaceae*, followed by a transition to *Firmicutes*-dominated ceca. Future studies will focus on understanding the functional and phylogenetic parameters of a normal developing microbiome, and to evaluate the effect of treatments like these on that normal range of microbial profiles.

## Conflict of Interest Statement

This research was funded in part through a gift from Star Labs Inc. (Clarksdale, MO, USA). Use of trade names in this publication does not imply endorsement by the North Carolina Agricultural Research Service or criticism of similar products not mentioned.

## Abbreviations

ANOSIM, analysis of similarity; DC, diluent-control; DP, diluent-probiotic; FDR, false discovery rate; KEGG, Kyoto Encyclopedia of Genes and Genomes; MANOVA, multivariate analysis of variance; OTU, operational taxonomic unit; PERMANOVA, Permutational MANOVA; PICRUSt, phylogenetic investigation of communities by reconstruction of unobserved states; PCoA, principal coordinate analysis; QIIME, quantitative insights into microbial ecology; ST, *Salmonella* Typhimurium; SIMPER, similarity percentage analysis; STAMP, statistical analysis of metagenomics profiles; VC, vaccine-control; VP, vaccine-probiotic.
