# Supplementary material for: Development of the Chick Microbiome: How Early Exposure Influences Future Microbial Diversity
Source: Front Vet Sci. 2016 Jan 20;3:2. doi: 10.3389/fvets.2016.00002 (PMC4718982; doi:10.3389/fvets.2016.00002)
Supplement: Supplementary file 1 [file table_1.docx]

**Supplemental Table 1. Diet Composition**

| **Feed component** | **%** |
| --- | --- |
| Ground Corn | 62.66 |
| Soybean Meal | 28.4 |
| Limestone | 1.2 |
| Dicalcium Phosphate (18.5% P) | 1.45 |
| Lysine HCl (78.5%) | 0.24 |
| DL-Methionine | 0.2 |
| Salt | 0.4 |
| Poultry TM (TM-90) | 0.2 |
| Choline Chloride (60%) | 0.05 |
| Vitamin Premix (NCSU-90) | 0.1 |
| Poultry Fat (Pet Food Grade) | 5 |
