# Supplementary material for: Development of the Chick Microbiome: How Early Exposure Influences Future Microbial Diversity
Source: Front Vet Sci. 2016 Jan 20;3:2. doi: 10.3389/fvets.2016.00002 (PMC4718982; doi:10.3389/fvets.2016.00002)
Supplement: Supplementary file 2 [file table_2.docx]

**Supplemental Table 2. Number of samples included in**

**analysis for each treatment and time-point**

|  |  | **Treatment** | | | |
| --- | --- | --- | --- | --- | --- |
|  |  | **DC** | **DP** | **VC** | **VP** |
| **Time-point** | **0** | 5^b^ | 4^b^ | 0^ab^ | 1^b^ |
|  | **1** | 5^b^ | 4^b^ | 5^b^ | 2^b^ |
|  | **3** | 6 | 6 | 5^b^ | 5^a^ |
|  | **7** | 5^b^ | 6 | 6 | 6 |
|  | **14** | 6 | 5^a^ | 6 | 5^b^ |
|  | **28** | 6 | 5^a^ | 6 | 6 |

^a^Samples were removed due to low sequence coverage

^b^Samples were unavailable or removed due to low concentration/quality

DNA
