# Supplementary material for: Development of the Chick Microbiome: How Early Exposure Influences Future Microbial Diversity
Source: Front Vet Sci. 2016 Jan 20;3:2. doi: 10.3389/fvets.2016.00002 (PMC4718982; doi:10.3389/fvets.2016.00002)
Supplement: Supplementary file 3 [file table_3.docx]

**Supplemental Table 3. Taxa removed from data set prior to PIRCRUSt analysis, day 14**

| **Order** | **Family** | **Genus** | **Vaccine-Probiotic** | **Diluent-Control** | **Vaccine-Control** | **Diluent-Probiotic** | **FDR P-value^a^** |
| --- | --- | --- | --- | --- | --- | --- | --- |
| Clostridiales | Other | Other | 0.041214068^b^ | 0.064510661 | 0.015479525 | 0.015402587 | 0.017 |
| Coriobacteriales | Coriobacteriaceae | Eggerthella | 6.04E-06 | 4.22E-06 | 0.00022303 | 0 | 0.017 |
| Clostridiales | Lachnospiraceae | Other | 0.011904706 | 0.008327372 | 0.022081509 | 0.00564618 | 0.017 |
| Clostridiales | Clostridiaceae | Other | 0.006184172 | 0.001117249 | 0.001332672 | 0.003856649 | 0.017 |
| Bacillales | Other | Other | 0 | 0 | 0 | 0.000271699 | 0.017 |
| Clostridiales | Ruminococcaceae | Other | 0.001114086 | 0.000300223 | 0.000643586 | 0.00058587 | 0.031 |
| Bacillales | Bacillaceae | Other | 5.08E-06 | 1.49E-05 | 0 | 0.015280536 | 0.072 |
| Bacillales | Bacillaceae | Bacillus | 0 | 0 | 0 | 0.001512628 | 0.072 |
| Bacillales | Planococcaceae | Other | 0 | 0 | 0 | 0.000587725 | 0.072 |
| Bacillales | Planococcaceae | Planomicrobium | 0 | 0 | 0 | 0.000115094 | 0.072 |
| Bacillales | Planococcaceae | Rummeliibacillus | 0 | 0 | 0 | 4.46E-05 | 0.072 |
| Turicibacterales | Turicibacteraceae | Turicibacter | 0 | 0 | 0 | 0.000108341 | 0.072 |
| Rickettsiales | mitochondria | Other | 6.04E-06 | 0 | 2.90E-05 | 0 | 0.072 |
| Thiotrichales | Thiotrichaceae | B46 | 0 | 3.17E-05 | 4.89E-06 | 0 | 0.072 |
| Lactobacillales | Other | Other | 0 | 1.84E-05 | 9.09E-06 | 0.000199248 | 0.072 |
| Clostridiales | Peptostreptococcaceae | Other | 0 | 7.43E-05 | 3.43E-05 | 0.000249114 | 0.072 |
| Other | Other | Other | 0.000492427 | 0.001540406 | 0.000857901 | 0.001166976 | 0.072 |
| Enterobacteriales | Enterobacteriaceae | Other | 0.002747429 | 0.002343169 | 0.001742816 | 0.000487024 | 0.114 |
| Lactobacillales | Enterococcaceae | Other | 2.77E-05 | 9.34E-06 | 3.32E-05 | 0.000111409 | 0.282 |
| Other | Other | Other | 1.22E-05 | 1.90E-05 | 0 | 0 | 0.419 |
| Other | Other | Other | 1.81E-05 | 0 | 0 | 0 | 0.493 |
| Rhizobiales | Brucellaceae | Other | 2.21E-05 | 0 | 0 | 0 | 0.493 |
| Lactobacillales | Leuconostocaceae |  | 0 | 1.49E-05 | 2.25E-05 | 0 | 0.509 |
| Clostridiales | Clostridiaceae |  | 3.02E-05 | 5.83E-06 | 5.17E-05 | 8.88E-05 | 0.509 |
| Bacteroidales | [Barnesiellaceae] | Other | 0 | 9.50E-06 | 0 | 0 | 0.509 |
| Other | Other | Other | 0 | 0 | 9.81E-06 | 0 | 0.509 |
| Clostridiales | Clostridiaceae | Sarcina | 0 | 1.17E-05 | 0 | 0 | 0.509 |
| Clostridiales | Lachnospiraceae | Lachnospira | 0 | 0 | 1.47E-05 | 0 | 0.509 |
| Other | Other | Other | 0 | 1.17E-05 | 9.09E-06 | 0 | 0.663 |
| Other | Other | Other | 0 | 9.92E-06 | 9.09E-06 | 0 | 0.663 |

^a^False-discovery-corrected P-values from Kruskal-Wallis tests.

^b^Values represent relative abundance as a proportion of 1.
