# Supplementary material for: Development of the Chick Microbiome: How Early Exposure Influences Future Microbial Diversity
Source: Front Vet Sci. 2016 Jan 20;3:2. doi: 10.3389/fvets.2016.00002 (PMC4718982; doi:10.3389/fvets.2016.00002)
Supplement: Supplementary file 4 [file table_4.docx]

**Supplemental Table 4. Taxa removed from dataset prior to PICRUSt analysis, day 28**

| **Order** | **Family** | **Genus** | **Diluent-Control** | **Diluent-Probiotic** | **Vaccine-Control** | **Vaccine-Probiotic** | **FDR P-value^b^** |
| --- | --- | --- | --- | --- | --- | --- | --- |
| Coriobacteriales | Coriobacteriaceae | Eggerthella | 0^b^ | 0 | 0.001462938 | 0.000180123 | 0.008 |
| Clostridiales | Other | Other | 0.108313679 | 0.052878584 | 0.043422939 | 0.012453526 | 0.011 |
| Enterobacteriales | Enterobacteriaceae | Other | 0.000790157 | 0.001483962 | 0.001049698 | 0.000143583 | 0.176 |
| Clostridiales | Clostridiaceae | Other | 0.003119151 | 0.001172701 | 0.00253042 | 0.002301304 | 0.276 |
| Clostridiales | Ruminococcaceae | Other | 0.002158875 | 0.00093703 | 0.00156716 | 0.00147708 | 0.276 |
| Enterobacteriales | Enterobacteriaceae | Citrobacter | 4.23E-05 | 1.01E-05 | 0 | 0 | 0.276 |
| Bacillales | Bacillaceae | Other | 0 | 0.000680933 | 0 | 1.12E-05 | 0.276 |
| Clostridiales | Lachnospiraceae | Butyrivibrio | 1.94E-05 | 3.10E-05 | 0 | 0 | 0.276 |
| Bacillales | Bacillaceae | Bacillus | 2.59E-05 | 0 | 0 | 0 | 0.280 |
| Clostridiales | Peptostreptococcaceae | Other | 6.82E-06 | 1.60E-05 | 3.80E-05 | 0.000155064 | 0.307 |
| Clostridiales | Lachnospiraceae | Other | 0.014636359 | 0.014745188 | 0.017493251 | 0.011349562 | 0.325 |
| Bacillales | Other | Other | 0 | 2.14E-05 | 0 | 3.22E-05 | 0.345 |
| Clostridiales | Clostridiaceae | Alkaliphilus | 0 | 0 | 0 | 9.41E-06 | 0.358 |
| Clostridiales | Lachnospiraceae | Lachnospira | 2.22E-05 | 1.78E-05 | 0 | 0 | 0.358 |
| Rickettsiales | mitochondria | Other | 3.59E-05 | 1.33E-05 | 4.71E-06 | 3.39E-05 | 0.450 |
| Other | Other | Other | 3.27E-05 | 9.05E-05 | 0 | 5.83E-05 | 0.458 |
| Other | Other | Other | 0 | 1.01E-05 | 0 | 0 | 0.458 |
| Rubrobacterales | Rubrobacteraceae | Rubrobacter | 1.36E-05 | 0 | 0 | 0 | 0.458 |
| Bacillales | Bacillaceae | Anaerobacillus | 3.31E-05 | 0 | 0 | 0 | 0.458 |
| Bacillales | Planococcaceae | Planomicrobium | 0 | 1.25E-05 | 0 | 0 | 0.458 |
| Lactobacillales | Other | Other | 0 | 2.14E-05 | 0 | 0 | 0.458 |
| Clostridiales | Lachnospiraceae | Clostridium | 0 | 1.60E-05 | 0 | 0 | 0.458 |
| Erysipelotrichales | Erysipelotrichaceae | Other | 1.36E-05 | 0 | 0 | 0 | 0.458 |
| Burkholderiales | Alcaligenaceae | Sutterella | 1.25E-05 | 0 | 0 | 0 | 0.458 |
| Desulfobacterales | Desulfobulbaceae |  | 0 | 1.07E-05 | 0 | 0 | 0.458 |
| Enterobacteriales | Enterobacteriaceae | Klebsiella | 1.36E-05 | 0 | 0 | 0 | 0.458 |
| Enterobacteriales | Enterobacteriaceae | Proteus | 0 | 1.60E-05 | 0 | 0 | 0.458 |
| Lactobacillales | Enterococcaceae | Other | 6.40E-05 | 3.74E-05 | 1.88E-05 | 1.50E-05 | 0.473 |
| Bacillales | Planococcaceae | Other | 0 | 0 | 0 | 7.62E-06 | 0.532 |
| Bacillales | Planococcaceae | Rummeliibacillus | 0 | 0 | 0 | 1.52E-05 | 0.532 |
| Clostridiales | Ruminococcaceae | Ethanoligenens | 0 | 0 | 0 | 8.82E-06 | 0.532 |
| Other | Other | Other | 1.23E-05 | 0 | 0 | 7.62E-06 | 0.583 |
| Clostridiales | Lachnospiraceae | Anaerostipes | 0 | 2.50E-05 | 0 | 8.82E-06 | 0.583 |
| Bacillales | Planococcaceae |  | 0 | 1.60E-05 | 0 | 1.90E-05 | 0.583 |
| Pseudanabaenales | Pseudanabaenaceae | Leptolyngbya | 1.32E-05 | 1.25E-05 | 0 | 8.82E-06 | 0.739 |
| Other | Other | Other | 0.001976425 | 0.00209155 | 0.001295505 | 0.001626973 | 0.840 |

^a^False-discovery-corrected P-values from Kruskal-Wallis tests.

^b^Values represent relative abundance as a proportion of 1.
